# Titanium Dioxide Nanoparticles in Food and Personal Care Products—What Do We Know about Their Safety?

**DOI:** 10.3390/nano10061110

**Published:** 2020-06-04

**Authors:** Joanna Musial, Rafal Krakowiak, Dariusz T. Mlynarczyk, Tomasz Goslinski, Beata J. Stanisz

**Affiliations:** 1 Chair and Department of Pharmaceutical Chemistry, Faculty of Pharmacy, Poznan University of Medical Sciences, Grunwaldzka 6, 60-780 Poznań, Poland; joanna.musial@student.ump.edu.pl; 2 Chair and Department of Chemical Technology of Drugs, Faculty of Pharmacy, Poznan University of Medical Sciences, Grunwaldzka 6, 60-780 Poznań, Poland; rlkrakowiak@gmail.com (R.K.); tomasz.goslinski@ump.edu.pl (T.G.)

**Keywords:** nanoparticles, titanium(IV) oxide, toxicity, titania, E171, exposure

## Abstract

Titanium dioxide (TiO_2_) is a material of diverse applications commonly used as a food additive or cosmetic ingredient. Its prevalence in products of everyday use, especially in nanosize, raises concerns about safety. Current findings on the safety of titanium dioxide nanoparticles (TiO_2_ NPs) used as a food additive or a sunscreen compound are reviewed and systematized in this publication. Although some studies state that TiO_2_ NPs are not harmful to humans through ingestion or via dermal exposure, there is a considerable number of data that demonstrated their toxic effects in animal models. The final agreement on the safety of this nanomaterial has not yet been reached among researchers. There is also a lack of official, standardized guidelines for thorough characterization of TiO_2_ NPs in food and cosmetic products, provided by international authorities. Recent advances in the application of ‘green-synthesized’ TiO_2_ NPs, as well as comparative studies of the properties of ‘biogenic’ and ‘traditional’ nanoparticles, are presented. To conclude, perspectives and directions for further studies on the toxicity of TiO_2_ NPs are proposed.

## 1. Introduction

### 1.1. Properties and Applications of Titanium Dioxide

Titanium dioxide (TiO_2_, titania, titanium(IV) oxide) is a material with a plethora of practical and possible applications. Commonly called ‘titanium white’, the fine white powder is mainly used as a pigment because of its brightness and opacifying strength (hiding power). TiO_2_ is resistant to chemical attack and displays excellent thermal stability, but most importantly, has the ability to both absorb and scatter the UV light (thanks to its high refractive index). These properties render the titanium pigment an irreplaceable ingredient in the production of paints, surface coatings, plastics, and paper [1]. The global production of titanium dioxide worldwide is continuously rising [2].

**Figure 1:**
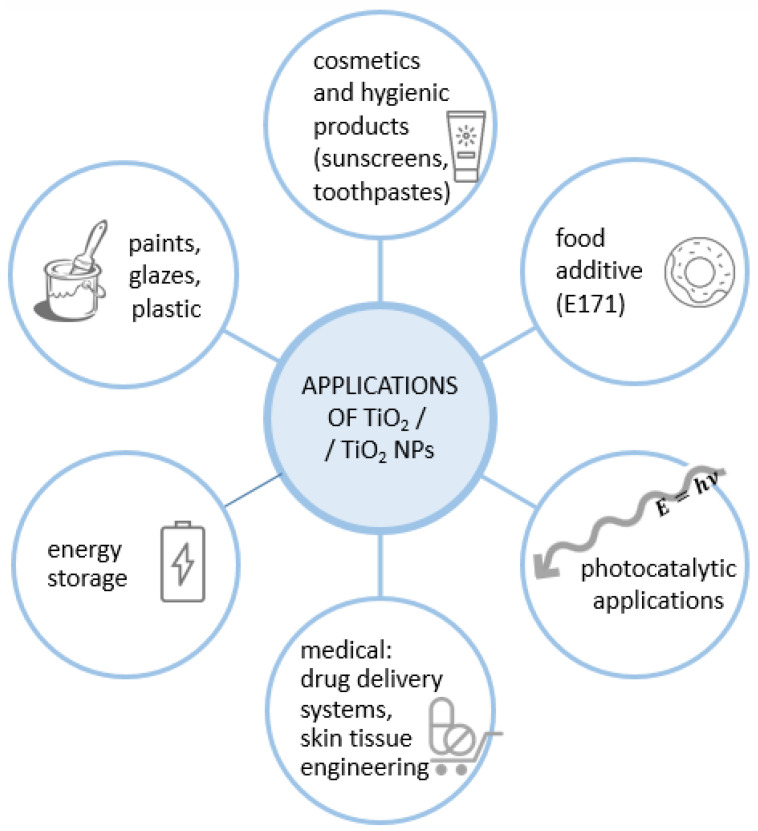
Various applications of titanium dioxide/titanium dioxide nanoparticles (TiO_2_/TiO_2_ NPs).

Titanium dioxide comes in three distinct crystal polymorphs—anatase, rutile, and brookite. Rutile is the most thermally stable polymorph, as both brookite and anatase are transformed into rutile when exposed to a temperature above 800 °C. All crystal forms of TiO_2_ offer photoactive properties. The differences in these properties can be characterized by different band gaps in TiO_2_ electron structures. Anatase was found the most photoactive form, as the bandgap, in this case, is higher compared to other polymorphs [1,3,4]. Manufacturing of the nanoscaled TiO_2_ particles, where at least one diameter is below 100 nm, has expanded the range of TiO_2_ utility (Figure 1). Titanium dioxide in the form of nanoparticles (TiO_2_ NPs) has become a common additive in paints, plastics, personal care products (cosmetics, sunscreens) and food—as the additive E171 [5,6]. Due to the properties which stem only from significantly decreased particle size (comparing between macro- or microparticles), titanium(IV) oxide nanoparticles are of great interest to many research groups [7]. In medical sciences, TiO_2_ was tested as a new effective drug carrier (for example, as TiO_2_ nanotubes) [8] or in skin tissue engineering and wound dressing [9,10,11]. Nanoscale TiO_2_ particles also have interesting photocatalytic properties, such as the ability to mediate photodegradation of pharmaceuticals, bacteria inactivation, the photooxidative killing effect on cancer cells, energy storage, as well as air and water purification [6,12]. Nowadays, TiO_2_ NPs are one of the most manufactured nanomaterials in the world [5,13].

Particles of size in the nanoscale have a higher surface-to-volume ratio, as compared to macro- or micro-particles. This fact affects their properties, such as reactivity of surface area, the degree to which the NPs aggregate, or bioavailability [14,15]. It is generally known that an increase in surface area accelerates the dissolution processes. Higher dissolution rates and smaller size of particles enhance their absorption through membranes [16], which leads to their deposition within tissues and organs after oral administration, while the insoluble material is mostly excreted with feces [17]. However, TiO_2_ has very low dissolution rate when compared to other metallic nanoparticles [18]. Brun et al. demonstrated that there was no visible dissolution of TiO_2_ particles for as long as 24 h after the uptake by human gut epithelial cells grown in in vitro monocultures [19]. As the dissolution rates achieved by TiO_2_ are very low [20], the cytotoxic effects caused by TiO_2_-NPs are more closely related to their size rather than due to metallic ions being released from the particles absorbed by cells. Such assumption was confirmed in the study by Gurr et al., where they demonstrated that very fine TiO_2_-NPs (<20 nm diameter) induced genotoxicity through oxidative stress in human bronchial epithelial cells, even without photoactivation of the nanomaterial [21]. Noteworthy, the same material sized >200 nm showed no sign of genotoxicity without irradiation.

If a substance additionally accumulates in biological tissues, its increased uptake may lead to adverse effects. This issue does not apply to larger forms of the same substance [14,22]. Upon introduction to biological systems, nanoparticles are exposed to a complex mixture of molecules, forming a so-called ‘corona’. This layer constitutes the interface between the nanomaterial and the environment and is often regarded as a biological identity of the particle. The corona plays a significant role in the bioactivity of a nanomaterial. It has been shown to mediate cellular responses (uptake, accumulation, intracellular localization, distribution and degradation) [23,24]. The protein corona (PC) is the most extensively studied nano–bio interface type [25]. With regard to titanium dioxide nanoparticles, Khan et al. recently assessed the impact of the surface chemistry on the behavior of the nanoparticle in an in vitro study, using adenocarcinomic human alveolar basal epithelial (A549) cells. Uncoated TiO_2_ NPs were compared with particles modified with PVP, Dispex AA4040, and Pluronic F127. The results revealed differences in terms of the tendency to form agglomerates, the rate of dissociation from corona proteins, dispersion of the particles and their degradation. Dispex AA4040, and Pluronic F127 coatings were found to influence the retention of PC and additionally exhibited an exchange between corona and intracellular proteins [26]. As the biocorona of the TiO_2_ NP notably affects its biological fate and therefore its potential toxicity, the studies on this interfacial layer should be included in the safety assessment of the nano-TiO_2_ used as food and cosmetic additive. These issues will be discussed later in this review.

### 1.2. Effect of TiO_2_ NPs Shape on Their Toxicity

So far, only a few studies have focused on the effect of the shape of the titania nanoparticles on their toxicity, mainly inhalation based, and much is yet to study on the subject. Allegri et al. compared the toxicity of TiO_2_ P25 nanoparticles with TiO_2_ nanofibers towards alveolar carcinoma epithelial cells [27]. The study concluded that although the nanoparticles exhibited a significant toxic effect, the nanofibers revealed a stronger impact on the tested cell viability and hemolysis. Worth noting is the fact that the TiO_2_ nanofibers caused more severe changes than P25 when either dose or surface area are taken into account. Additionally, nanofibers induced similar inflammatory response as crocidolite, a known cancer-inducing mineral. Such results correlate well with the study by Porter et al. in which mice exposed to different titania nanoparticles by inhalation showed more significant lung damage and inflammation in the case of nanobelt-shaped particles when compared to nanospheres [28]. These effects were linked to the length of the particles, as longer nanobelts induced a stronger response. Similar results, pinpointing the anatase nanobelts as more hazardous after inhalation compared to P25 nanospheres and anatase nanospheres, were also reported [29]. In another study, it was found that differently shaped anatase nanoparticles (nanotubes, nanocubes, nanospheres) caused similar effects when dosing was based on the surface area of the materials [30]. However, the nanotubes were associated with the alveolar proteinosis and occurrence of the inflammatory response. On the other hand, titania nanotubes were found to be more cytotoxic than P25 only at the concentration of 2.5 µg/mL when tested on cardiomyocytes in vitro [31], with much higher internalization (by diffusion and endocytosis) into the cells. A comparison of P25 and food grade titania with TiO_2_-based bipyramids, rods and platelets indicated that only the food grade titania and platelets were genotoxic to human epithelial cells in vitro [32]. TiO_2_ nanorods demonstrated a dose-dependent toxicity in alveolar adenocarcinoma cells in vitro but were not compared to differently shaped TiO_2_ nanoparticles [33]. In the same study, the rats that inhaled the nanorods were found to exhibit tissue damage, acute and chronic lung inflammation, and increased levels of titanium were measured not only in lungs but also in the bloodstream. Simon et al. attempted to find a relationship between toxicity and shape of the used titania nanoparticles [34]. In this study however, it was found that different nanoparticles were decreasing cell viability in a different manner, depending on the cell line used. Human umbilical vein endothelial cells (HUVEC cells) were affected by P25 spheres, sol-gel based isotropic nanoparticles and nanosheets, with no effect observed when nanoneedles were used. HEKn cells were most affected by nanosheets and in a smaller manner by P25 spheres, sol-gel based isotropic nanoparticles and nanosheets. Interestingly, HeLa cell proliferation was decreased slightly at high doses of sol-gel based isotropic nanoparticles and nanosheets but not by P25 nanospheres or nanoneedles.

All these cited studies indicate that the shape of the particles plays an important role in the toxicity of the titania nanoparticles. The biological effects observed for various TiO_2_ NPs are enhanced by the elongated form (tubes or fibers). Their increased internalization to the cells results in higher accumulation, which in turn explains its hampered clearance from the lungs.

### 1.3. The Role of Oxidative Stress in the Toxicity of Nanoparticles

**Figure 2:**
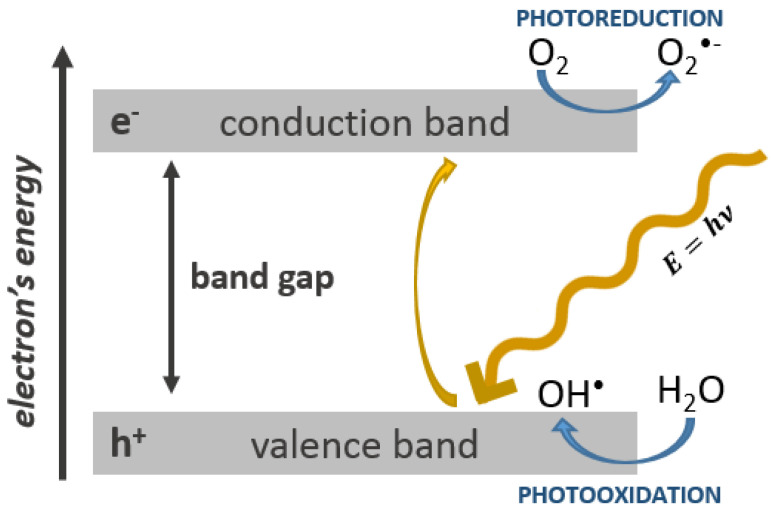
Bandgap in a semiconducting material. A valence band electron (e^−^) is excited to the conduction band upon light absorption (of ≥ bandgap energy) and leaves a hole in the valence band (h^+^) (according to [4,13]).

There is a plethora of studies that associate cyto- and geno-toxicity with their photocatalytic activity [4,13]. As mentioned before, TiO_2_ NPs can both scatter and absorb the UV radiation. UV light absorption is possible due to the semiconducting properties of TiO_2_ (Figure 2). The electrons from the valence band are promoted to the conduction band which photogenerates holes in the valence band. These holes and electrons can recombine or migrate to the NP surface where different redox processes take place, which causes reactive oxygen species (ROS) production.

**Figure 3:**
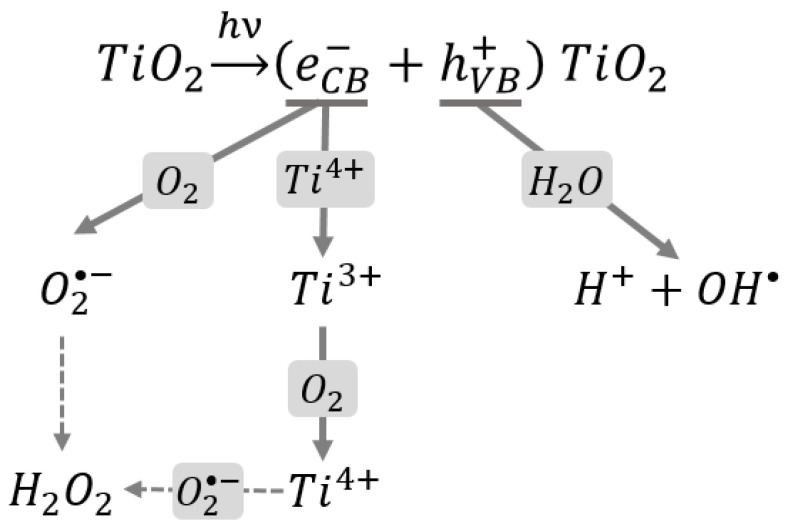
Photo-excitation of TiO_2_ and generation of reactive oxygen species (according to [4,13]).

The valence band holes react mainly with the moisture on the surface of particles, which results in the production of hydroxyl radicals. However, the conduction band electrons can interact with oxygen molecules (also present on the surface of particles) or be captured at Ti^IV^ sites, and later react with oxygen. As a consequence, it leads to the formation of hydrogen peroxide and superoxide anion radicals (Figure 3). All of the products mentioned above, such as hydroxyl radical, hydrogen peroxide, and superoxide anion radical, constitute a group of reactive oxygen species, which may impair the cell function [4,13,35].

Regarding the molecular mechanisms of the in vivo toxicity of NPs in general, the oxidative stress plays the most crucial role. For example, Nel et al., in their study, presented a direct relationship between the surface area, ROS-generating capability, and proinflammatory effects of nanoparticles in the lung [22]. ROS generated by mitochondria in cells are normally quickly neutralized by antioxidant substances. However, an excessive generation of oxidants, such as ROS, causes an imbalance between oxidants and antioxidant processes, which is called oxidative stress [14,15,22,36]. Oxidative stress has been proven to contribute to many types of human chronic diseases, such as cancer, as well as inflammatory, neurodegenerative or cardiovascular diseases [37]. According to Aillon et al., the organs that are the most exposed to oxidative stress are the liver and spleen, due to the accumulation of NPs capable of generating ROS. In this regard, high blood flow and slow clearance make kidneys and lungs very vulnerable to oxidative stress [14].

Taking into consideration these properties and the prevalence of TiO_2_ NPs in the products of everyday use, the recently emerging concerns seem to be understandable. Although a considerable amount of literature on the toxicity of TiO_2_ NPs is available nowadays—including excellent reviews by Skocaj et al. [38] and Shakeel et al. [39]—there is still much uncertainty, as some findings are inconsistent. Moreover, it is difficult to establish the intake levels of TiO_2_ NPs, as they depend on the type of the product consumed or used, its formulation, route of exposure, and the rate of consumption or usage of the product by a person [40]. The purpose of this paper is to review recent reports on the toxicity of titanium dioxide nanoparticles as food and cosmetic additives, to systematize these findings, and to point out perspectives for their further development.

## 2. Routes of Exposure and Toxicity of TiO_2_ NPs

**Figure 4:**
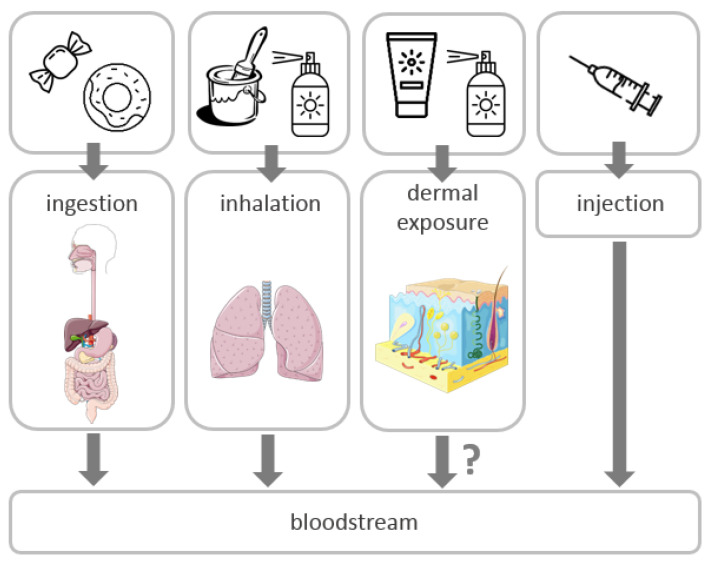
Routes of exposure and distribution of TiO_2_ NPs in the human body.

It is now a well-established fact, based on a variety of studies, that there are four main routes of exposure to titanium dioxide nanoparticles in humans: ingestion, pulmonary absorption (mainly through inhalation), dermal exposure and injection (Figure 4) [39].

Researchers agree that the ingestion, inhalation, and injection of TiO_2_ NPs lead to their systemic disposal. However, in case of dermal exposure, the findings are inconsistent. The following section of this paper attempts to indicate the main uncertainties related to the toxicity of TiO_2_ NPs as additives in food and cosmetic products, which are in the review of current chemical and toxicological studies.

### 2.1. Ingestion—TiO_2_ NPs as a Food Additive (E171)

In the food industry, TiO_2_ has been applied as an additive to enhance the white color of certain products, such as sweets or milk-based products [6,41,42]. In 2012, Weir et al. measured and compared the amount of titanium in common food products [6]. The obtained data, normalized to the titanium per serving, proved that the highest titanium contents could be found in chewing gums, candies, powdered sugar toppings, or products with white icing. The difference in TiO_2_ consumption between women and men in the United States was negligible. However, the difference in consumption between children and adults was found significant. Children are susceptible to consume up to four times more TiO_2_ per kilogram of body weight (kgbw) than an adult person. This fact can be simply explained by their consumer preferences, generally based on the taste in sweet snacks, among which many contain E171. Therefore, exposure to TiO_2_ depends also on dietary habits [6].

As the daily exposure to E171 may reach several hundreds of milligrams of which a considerable part appears in the nano range (about 36%) [6], there are concerns that a long-term exposition to this substance may lead to harmful effects in the human body. In response to this growing public worldwide health problem, the European Food Safety Authority (EFSA) published a ‘Re-evaluation of titanium dioxide (E171) as a food additive’, based on documentation on usage levels and safety of titanium dioxide provided by various international associations, councils and committees. The EFSA panel concluded that both the absorption and the bioavailability of orally administered micro- and nano-TiO_2_ is low. Most of the TiO_2_ ingested dose is eliminated unchanged in the feces, except for a tiny amount (not exceeding 0.1%), which is absorbed by the gut-associated lymphoid tissue and distributed to various organs. The panel stated that the micro- and nano-sized particles are unlikely to cause a genotoxic hazard in vivo [43]. However, a year later, a study conducted by Bettini et al. [44] proved that orally administered food-grade TiO_2_ containing nanoscale particles impaired immune homeostasis and induced carcinogenesis in rats. Based on this publication, the French ANSES (Agency for Food, Environmental and Occupational Health) published their opinion on TiO_2_ NPs [45], in which the necessity of conducting thorough research on the possible dangers connected with the usage of E171 was underlined. France is the first country to ban using the E171 food additive because of the possible harmful effects on humans and a lack of scientific data to confirm its safety. The restrictions became effective in 2020 [46].

The genotoxic potential of E171 has already been proven in several studies. In 2016, Proquin et al. [47] used an in vitro model with human Caco-2 and HCT116 cells to research the potentially toxic effects of E171, containing fractions of micro- and nano-particles (MPs and NPs, respectively). Their findings proved the highest capability to induce ROS generation in a cell-free environment for E171 (defined as a mixture of 39% TiO_2_ NPs and 61% MPs), followed by NPs and MPs. However, in a cellular environment, only MPs revealed the capacity to produce ROS, which, as they suggested, can lead to a proinflammatory response. On the other hand, the NPs did not stimulate ROS production, which was explained by the fact that following internalization, they react with cellular structures blocking ROS formation. This study also provided evidence for single-strand DNA breaks in Caco-2 cells induced by all E171, NPs, and MPs. The researchers suggested that the E171 was more toxic to Caco-2 cells than NPs or MPs alone. Harmful effects of TiO_2_ NPs were also confirmed by Grissa et al. [48]. The assumption of this study was to simulate long-term, low dose ingestion of E171 in humans. For this purpose, anatase NPs (5–12 nm in size) were administered intragastrically to Wistar rats for 60 days. As a result of the performed study, there were noted changes in the hematopoietic parameters, as well as a genotoxic effect of TiO_2_ NPs in vivo at 100 and 200 mg/kgbw. On the other hand, the particles that were used in this study were generally smaller than those found commonly in foodstuffs [6], and the crystalline phase of anatase is known to be much more toxic than rutile. In a study by Talamini et al., a material exhibiting foodstuff-grade particle size distribution was used [49]. The researchers studied a repeated 3-week oral administration of E171 to mice (E171 suspension dripping into the mouth of mice, 5 mg/kgbw for 3 days per week). The results were related not only to toxic outcomes, such as an inflammatory response and increased superoxide production in the digestive tract, but also to the deposition of TiO_2_ in the internal organs, especially in the liver and large intestine, where a three-fold increase in TiO_2_ NPs was noted [49].

Oral exposure to TiO_2_ NPs is associated not only with the ingestion of E171, but also with the consumption of pharmaceuticals. TiO_2_ is a common pharmaceutical excipient, mostly used as a white pigment, but in its nanoform, it can also be an effective carrier of antibiotics, which additionally enhances or prolongs the action of the drug [50,51,52]. Evidence for genotoxic effects of nano-TiO_2_ drug carrier administered orally was recently provided by Mottola et al., who researched the influence of nano-TiO_2_ and lincomycin coexposure on human amniocytes. The results of this in vitro study demonstrate that the exposure to TiO_2_ NPs induced an increase in DNA strand breaks, a loss of DNA stability and apoptosis, as well as reduced cells viability, whereas the exposure to lincomycin itself had no toxic/genotoxic effects on amniotic cells. The authors suggested that the underlying molecular mechanism of the DNA damage may be the production of ROS by the NPs, notably the ^●^OH radical [50,53]. To date, researchers usually associate the genotoxicity of TiO_2_ NPs with the formation of oxidants [54,55,56,57,58,59].

Toothpaste is another source of TiO_2_ (also in a nanoform) which may be ingested. Therefore, it is not surprising that attention has now turned to this personal care product. The review of scientific data on this subject, which was carried out by national and international agencies, led to a prohibition of E171 usage in food production [60,61]. Usually the amount of toothpaste used is small, so the ingestion of TiO_2_ NPs is possible only in case of unwanted swallowing. Thus, taking into consideration the low absorption of TiO_2_ administered orally, the appearance of toxic effects is rather unlikely [43].

As mentioned earlier, nanomaterials can interact with molecules, which are present in biological fluids, for example, bacterial lipopolysaccharide (LPS), which is a proinflammatory compound present in the gastrointestinal tract. Bianchi et al. indicated that LPS included in the biocorona of the titania P25 particle displays enhanced proinflammatory effects [62]. The biological fate of nanomaterials should be also evaluated with regard to food ingredient effects. As an example, model food ingredients, bovine serum albumin and sucrose were able to stabilize TiO_2_ NPs and induced a decrease in their agglomerate sizes [63]. It has been also shown that the adsorption of proteins on the food grade TiO_2_ nanoparticles is inhibited in the presence of oxalate, a dicarboxylic acid, or phosphates [64]. As TiO_2_ NPs are largely utilized in dairy-based products, Cao et al. focused on their interactions with milk proteins. The researchers observed dissociation of casein micelles and formation of NP-protein complexes. It was suggested that this interaction may have altered the shielding of the peptide bonds. Therefore, it could be supposed that the amount of undigested protein, which may reach the colon and affect the intestinal microflora, would be significantly changed [65].

### 2.2. Local Effects of Tio_2_ NPs on the Intestinal Barrier and Changes in the Gut Microbiota

The safety assessment of the food-grade nano-TiO_2_ should be also regarded from the perspective of the local effects that may appear. This issue should not be omitted, because even if the TiO_2_ NPs may not provoke toxic effects due to their penetration, local damages in the gastrointestinal tract may disrupt the essential nutrient absorption. An overview of studies from the last six years shows a consensus among researchers on the detrimental effects caused by TiO_2_ NPs, both in vitro and in vivo. In 2014, Botelho et al. conducted a study on human gastric epithelial cells and stated that titania NPs provoked tumor-like phenotypes. Briefly, they observed an increase in the proliferation of the cells and a decrease in their apoptosis. They also detected increased glutathione levels, which is a sign of oxidative stress-mediated toxicity, as well as DNA lesions [66]. Urrutia-Ortega et al. observed that intragastric E171 exposure increased tumor progression markers (COX2, Ki67 and β-catenin included) and enhanced tumor formation in the distant colon in a murine model [67]. They noted that TiO_2_ did not induce tumor formation itself, but led to dysplastic changes in colonic epithelium and a decrease in goblet cells. Moreover, they concluded that the exposure to E171 may worsen pre-existing intestinal disorders. This was confirmed by Ruiz et al., who noticed an aggravation of acute colitis in a mouse model following oral gavage, as well as accumulation of titania crystals in the spleen. Moreover, the in vitro experiments proved that the particles were taken up by the human epithelial cells and macrophages and activated the NLRP3 inflammasome. Additionally, after the assessment of titanium levels in blood samples from human volunteers, they discovered increased titanium levels in samples from patients with ulcerative colitis, compared with healthy donors and patients with inflammatory bowel disease [68]. An important conclusion in this case is that the exposure to E171 is strongly contraindicated in patients with pre-existing inflammatory conditions or an impaired intestinal barrier function. A few recent experiments on a well-established cell line Caco-2 support former studies, indicating detrimental effects on the intestinal epithelium layer. Taken together, the results point out that exposure to TiO_2_ NPs has the following local effects:

- induces an inflammatory response [69,70,71,72];
- increases the release of mucins and the expression of some efflux pumps [69];
- increases ROS generation [70];
- induces morphological changes or decreases the number of intestinal microvilli, which in turn decreases the surface area needed for optimal nutrients absorption [70,72,73];
- leads to their internalization and entrapment by Caco-2 monolayers [71].

Another interesting explanation for the local toxicity of TiO_2_ NPs has been recently suggested by Yao et al., who remarked that it may be caused by an imbalance between the Th1 and Th2 cells, resulting in the tight junction barrier damage [73].

As far as proper intestinal function is concerned, the importance of the gut microbiota cannot be ignored. The human gut microbiome is a complex ecosystem and its imbalance may lead to pathogenesis or progression of a large spectrum of diseases [74]. Recent studies, both in vitro and in vivo, provide an insight into the influence of nano-TiO_2_ on the gut microbiota. Dudefoi et al. employed a defined human gut bacterial community, microbial ecosystem therapeutic-1 (MET-1) to evaluate the impact of two food-grade TiO_2_ additives. MET-1 contains 33 bacterial strains which can be cultured as an ecosystem. The researchers did not observe a significant alteration of the human gut microbiota, however, they were concerned about the cumulative effects of chronic ingestion of the nanoscale titania [75]. A limited impact on microbial communities has also been observed by Agans et al. [76]. Alterations in intestinal microbiota composition were noted by Radziwill-Bienkowska et al. [77]. They presented the data on the changes occurring in response to some factors such as intestinal disorders, diet variations and microbial challenges. In turn, Pinget et al. performed an in vivo study in mice and confirmed that food grade TiO_2_ had minimal influence on the gut microbiota composition. In addition, they found that it can still significantly impair the gut homeostasis. The impact of TiO_2_ included colonic inflammation, increased inflammatory response and altered release of bacterial metabolites [78]. Chen et al. also associated the disorders of gut microbiota with an inflammatory response and suggested that the oxidative stress may contribute to the underlying mechanism [79]. Overall, further investigation is needed to determine the effects of chronic exposure to the food-grade TiO_2_, particularly in vulnerable subpopulations.

### 2.3. Dermal Exposure—TiO_2_ NPs as a Sunscreen Compound

Sunscreens are another type of commonly used personal care products with a relatively high content of TiO_2_ NPs. Formulations with nanoscale TiO_2_ are useful in terms of light scattering and UV absorption. Moreover, when applied on the skin, they look more transparent, which is a desirable property for many consumers [13,80]. Although TiO_2_ NPs in sunscreens have already been studied for nearly two decades [81], some questions and uncertainties remain still unresolved, and regulation of the usage and safety of TiO_2_ NPs in these products is needed [82].

As mentioned before, smaller particles are more effective in terms of light scattering and absorption. However, the small size also increases possible absorption through the skin. It has not yet been determined which size of titania nanoparticles in sunscreen provides the best protection against UV radiation. Because the ozone layer almost entirely absorbs the UV-C radiation, skin should be protected in the UV-B (290–320 nm) and UV-A (320–400 nm) regions. More insight into this topic was given by Popov et al., who tested TiO_2_ NPs of six different sizes for their ability to stop the 307–311 nm light [83]. The study performed on six healthy volunteers with the so-called tape-stripping technique was applied keeping a proper timeline in order to assess the in-depth distribution of the fine TiO_2_ particles. Scattering and absorption coefficients for a medium containing TiO_2_ particles of different volume concentrations were calculated using Monte Carlo simulations. The Monte Carlo method was also developed to simulate UV-B propagation within the horny layer containing the embedded TiO_2_ particles. The results obtained in their study indicated that TiO_2_ NPs of 62 nm diameter revealed the optimal protective properties. Interestingly, the diameter of 62 nm was neither the smallest nor the largest one tested. Additionally, the researchers experimented on the concentration of TiO_2_ in subsequent layers of the stratum corneum, which revealed that titania could be found even 15 µm deep [83].

To assure effective protection against UV radiation, it is essential to determine not only the size of TiO_2_ particles, but also their shape. To date, several studies have conducted a thorough analysis of TiO_2_ particles extracted from a sunscreen formulations [84,85,86]. Interesting results were recently reported by Ilić et al., who evaluated the in vitro effect of TiO_2_ nanomaterials of three morphologies on human keratinocytes (HaCaT). They discovered that nanowires and nanoplates were significantly more effective in protecting human skin cells from UV-B induced damage. It can be concluded that TiO_2_ NPs can be designed specifically in order to enhance the quality and efficacy of a sunscreen product [87].

It is essential to thoroughly control the sunscreen formulations in order to verify the size of the NPs, their size distribution, aggregation rate, and the concentration of the NPs. According to the opinion of the Scientific Committee on Consumer Safety of the European Commission (SCCS), TiO_2_ NPs used in sunscreens up to a concentration of 25% can be considered to not pose any risk of adverse effects in humans after application on healthy, intact or sunburnt skin [88,89]. The parameters of NPs ought to be thoroughly controlled to ensure complete safety of usage for every sunscreen product. Although a few national and international institutions have proposed recommendations for labeling sunscreen products and testing their effectiveness [89,90,91,92,93], there is still a lack of official guidelines for a thorough characterization of TiO_2_ NPs in sunscreen formulations, which would set the standards for the quality control methods. Recently, the U.S. Food and Drug Administration (FDA) has issued a rule in order to update the regulatory requirements and put into effect the final monograph for over-the-counter sunscreen drug products. This rule applies to sunscreens marketed without FDA-approved applications. The FDA proposes to mark two ingredients, TiO_2_ and ZnO, as ‘generally recognized as safe and effective’ [94].

Recent literature highlights some modern techniques of the physicochemical characterization of nanosized TiO_2_, which could be implemented in common industrial practice through official guidelines [80,95,96]. Contado and Pagnoni presented flow field-flow fractionation (FlFFF) combined with inductively coupled plasma-atomic emission spectrometer (ICP-AES) as a relatively simple, low-cost, yet powerful tool for determining the TiO_2_ content and particle-mass size distribution (PSD) in sunscreen lotions [96]. A recent study by Bocca et al. used ICP-MS (inductively coupled plasma-mass spectrometry) and its modification SP ICP-MS (single particle inductively coupled plasma-mass spectrometry) to determine and compare the concentration and particle size distribution of TiO_2_ NPs in commercial sunscreens [80]. In that study, ICP-MS was used both as a direct technique, SP ICP-MS, and as a detector combined with the asymmetric flow-field flow fractionation (AF4-FFF), for preseparation, on-line coupled to the multi-angle light scattering (MALS). The results of that study indicated that the concentration of TiO_2_ NPs in creamy applications did not exceed the SCCS limit of 25%, and therefore, their usage can be considered safe [80]. Despite this, in 2001, Serpone et al. cast doubt upon the biological safety of the TiO_2_-containing sunscreens [81]. In the presence of fine TiO_2_ particles, they observed their harmful effects on DNA after illuminating supercoiled plasmids with simulated sunlight. The researchers also tried to fabricate photocatalytically inactive TiO_2_ specimens by modifying the particle surface. It should also be noted that the deleterious effects of TiO_2_ on DNA were possible due to the penetration of these NPs through the cell membranes. This issue remains a matter of argument. Thus far, several studies have reported that the TiO_2_ NPs do not cross the stratum corneum (SC), the outermost epidermal layer, and that the number of the particles passing through the SC is insignificant. SC is generally an effective barrier against the transfer of chemicals through the skin. It consists of dead cells incapable of active transport of substances. It has been already shown that after a two-hour exposure to sunscreens containing TiO_2_ and ZnO NPs, their levels in human viable epidermal layers were too low to be tested [97]. Thus, these results confirmed that the penetration through the SC is unlikely. Another study, in which sunscreen formulations containing 5% TiO_2_ (coated and noncoated NPs) were applied topically to Yucatan minipigs, also reported no significant penetration through normal, unharmed skin [98].

In contrast to the findings mentioned above, some researchers claim that nanosized TiO_2_ can penetrate the skin and induce tissue damages, even in major organs. An in vitro and in vivo study by Wu et al. demonstrated that TiO_2_ NPs do not pass through the SC of isolated porcine skin after 24 h exposure, but after 30 days of topical application, the NPs were found in deeper layers of the epidermis [99]. Moreover, subchronic (60 days) dermal exposure in hairless mice proved that TiO_2_ NPs could not only penetrate through the SC but also reach different tissues and induce pathological lesions, among which the most severe ones were displayed in the skin and liver. The authors also detected an elevated malondialdehyde level and a decreased superoxide dismutase level, which proved that these NPs induce oxidative stress processes. In conclusion, they also stated that TiO_2_ NPs topically applied on skin for a prolonged time can induce skin aging [99]. A very recent experiment by Pelclova et al. confirmed that TiO_2_ NPs could penetrate skin [100]. Detectable levels of nano-TiO_2_ were found in blood and urine of the human volunteers up to one week after using the sunscreen formulation. Furthermore, it was found that although the TiO_2_-based sunscreens prevented sunburns, they did not decrease the systemic oxidative stress, as evaluated by the tested biomarkers.

The results of various dermal exposure studies, both confirming and disproving the penetration of TiO_2_ NPs through the SC, are summed up in Table 1.

A considerable amount of studies have also examined the influence of TiO_2_ NPs on human cell lines in in vitro experiments. For the human keratinocyte cell line HaCaT (human adult low calcium high-temperature keratinocytes), following exposure to TiO_2_ NPs, decreased cell viability and induction of the cell cycle arrest have been demonstrated [110]. Rutile TiO_2_ NPs with <100 nm particle size were also tested on a human metastatic melanoma cell line, where a reduction in cell metabolic activity and cytotoxic response were observed. Especially interesting was a study of the influence of nano-TiO_2_ on the expression of mRNA of the ABCB5 transmembrane protein. The researchers presented that the studied nanomaterial might influence cell invasiveness and aggressiveness as the protein ABCB5 is closely linked to tumorigenicity, progression, and disease recurrence of some human malignancies [111].

The debate continues also on the potential penetration of titania NPs through damaged skin. Sunscreens are often applied on skin which is already sunburnt, dried out by UV irradiation, affected by beauty procedures (e.g., hair removal) or irritated by environmental factors (wind, salt and sand). In general, it should be noted that any changes in the composition of lipids caused by skin damages may impair the barrier function of the skin and therefore facilitate the penetration of NPs [4,112]. To date, the results of most comparative studies, both for commercial sunscreen formulations and nano-TiO_2_ suspensions, indicate that slight skin damages do not enhance its permeability [102,103,104,105,106]. However, it has to be emphasized that sunscreens definitely should not be applied on mechanically injured skin or an open wound. Besides, many authors have remarked that sunscreens are often used in sprayable forms, and this way of application may cause potential health risks in another manner—by inhaling TiO_2_ NPs. A variety of sunscreens is available in such a form. This issue concerns emulsions or oil sprays, foams, as well as mists. Sprayable forms have become increasingly common among consumers because of their ease of use. Inhalation exposure to TiO_2_ has been evaluated in several epidemiological analyses [113,114,115,116,117,118]. In these studies, as well as in case reports on human exposure to inhaled TiO_2_ [119,120], it has conclusively been shown that there is no positive correlation between the occurrence of carcinogenic effects and the occupational exposure to titania. However, it has to be emphasized that most of these studies provided no indication on the size of TiO_2_ particles.

Taking this into consideration, the International Agency for Research on Cancer (IARC) stated that the exposure to titanium dioxide is not directly associated with an increased cancer risk. Nevertheless, after assessment of the data derived from animal model studies, the IARC decided that there exists sufficient evidence to claim carcinogenicity of titanium dioxide to animals [1]. However, these data must be interpreted with caution, as various methodological approaches were adopted. Experiments concerned both the micro- and the nano-form of titanium dioxide. What is more, the results cannot be easily extrapolated to humans, because concentrations employed in some cases exceeded maximum human exposure. Overall, the IARC includes TiO_2_ in the group of substances which are possibly carcinogenic to humans (Group 2B). The ongoing discussion about the potential deposition and toxic effects of the nanoparticles caused by their inhalation needs to be resolved. Currently, the IARC advises against using sprayable sunscreen products. It should also be remarked that children are particularly susceptible to an unintended inhalation of TiO_2_ NPs since many sunscreen formulations for children come in the form of a spray or a foam, as these methods render the formulation easier to dispense and spread.

Recently, the attention of researchers studying TiO_2_ toxicity in sunscreens has turned to the surface and the entourage of TiO_2_ nanoparticles. For example, Y_2_O_3_-decorated TiO_2_ nanoparticles were found to display enhanced UV attenuation and reduced photoactivity and consequently, cytotoxicity, compared with a commercial TiO_2_ sample. The authors suggested the inclusion of these materials into sunscreen products [121]. In another study, coating of TiO_2_ NPs with dihydroxyphenyl benzimidazole carboxylic acid (Oxisol) not only led to photolytic activity reduction, but also boosted its antioxidant effects and stabilization of the formulation [122]. By modifying the surface of TiO_2_ NPs, it is also possible to improve the appearance of a sunscreen formulation, as formulations containing TiO_2_ NPs modified with a complexing compound, *p*-toluene sulfonic acid, were found to be more transparent [123].

The aforementioned issue of the protein corona should also be considered regarding the safety of nano-TiO_2_ as a sunscreen formulation compound. Serum proteins around the surface of the NP may undergo oxidation, even upon low generation of ROS, which provokes an oxidative stress response [124]. Future development of sunscreen ingredients should therefore comprise a proper design of their chemical surface. Furthermore, Sanches et al. stated that different contents of proteins, as well as other molecules (such as calcium or phosphorus) present in the biological medium, conceal TiO_2_ NPs and may influence their uptake and distribution [125]. An important remark is that a thorough analysis of TiO_2_ nanomaterial for sunscreen products should be performed also with regard to the nano–bio interactions. Additionally, Filipe et al. suggest that ROS or lipid peroxidation products appearing on the surface of the skin are prone to diffuse underneath the SC and subsequently lead to oxidative damage [97]. Moreover, ROS generated by nano-TiO_2_ may affect the transformation of other commonly used compounds of sunscreen formulations, including parabens, and increase their bioavailability and toxicity [126]. Several studies postulated that extreme stability and very poor aqueous solubility of TiO_2_ [127] could render its insolubility in sunscreens, making it biologically inert [13]. Nevertheless, some sunscreen formulations contain hydroxyacids (for example, citric or salicylic acid) which have the ability to chelate Ti^IV^, leading to its dissolution [13].

Regarding dermal exposure of TiO_2_ NPs on human skin, it should be underlined that cream formulations containing these nanoparticles also reveal an impact on human cutaneous bacteria strains. Interestingly, this influence highly depends on the surface properties of NPs, mostly changes in polarity and charge, but also on the timescale of emulsions aging [128].

## 3. ’Green’ TiO_2_ NPs—Safer Perspective for the Future?

Numerous studies have already demonstrated that various metallic and metallic oxide NPs may be fabricated in compliance with green chemistry assumptions. ‘Green synthesis’ is often preferred over traditional methods for its many advantages, such as effectiveness, eco-friendliness, ease of characterization, fewer chances of failure, fast performance, and low cost [129]. It has been suggested that the materials used to fabricate the NPs greatly influence their morphology and physicochemical properties, which may have an impact on their further utilization [129]. ‘Green synthesized’ NPs, often called ‘biogenic NPs’ are generally considered safe, and in some cases, they display better properties to those synthesized with ‘traditional’ methods [130,131,132]. Currently, several methods have been developed for the synthesis of green NPs. Some of those technologies include the use of vitamins, like vitamin B2 or ascorbic acid, as well as enzymes from various plant extracts. They are in accordance with bio-based methods, which may involve the use of plants, bacteria, fungi or algae [129].

A considerable amount of studies has already described the green synthesis of TiO_2_ NPs and their characterization. Different approaches towards fabrication techniques of biogenic TiO_2_ NPs have been demonstrated and summed up in recent reviews and are beyond the scope of this paper [133,134]. However, compared with the amount of publications on various synthesis methods for ‘green’ TiO_2_ NPs, a relatively small body of literature touches upon their properties and compares their effectiveness with chemically derived TiO_2_ NPs [135,136,137]. For instance, there were published studies pointing out their antimicrobial activity. TiO_2_ NPs prepared with the use of *Hibiscus rosa sinensis* flower extract displayed not only significant activity against pathogenic bacteria but also acted more effectively than those synthesized by chemical synthesis [134]. In another study, ‘green’ TiO_2_ NPs, obtained by rapid synthesis using *Moringa oleifera* aqueous leaf extract, were found to exhibit significant wound healing activity in rats when compared with standard antibiotic drug for treatment of local infections [137]. A different approach was undertaken by Yu et al., who used lignosulfonate (LS), a natural macromolecular sun-blocker, to modify the surface of TiO_2_ NPs and therefore enhance the UV-blocking efficiency of the nanoparticles. Sunscreens containing TiO_2_@LS nanocomposites exhibited 30–60% higher SPF values than creams with the same dosage of nanograde TiO_2_ [138].

Taken together, these findings recommend the employment of ‘green’ TiO_2_ NPs for dermal applications. Continued efforts are needed to implement the use of safe and eco-friendly TiO_2_ NPs into sun-blocking formulations.

## 4. Conclusions and Perspectives

This paper has raised important questions on the current state of knowledge on the toxicity of titanium dioxide nanoparticles—a chemical compound commonly used in various everyday applications. In general, current findings seem to be inconsistent and highlight the necessity of establishing safety recommendations for TiO_2_ in its nanoform, regarding its applications as a food additive and cosmetic ingredient (Table 2).

There are few long-term exposure studies on the safety of TiO_2_ NPs usage as a food additive or a sunscreen ingredient. Despite the EFSA statement that the absorption of TiO_2_ after oral administration is extremely low [43], the usage of this ingredient, especially in nanoform, raises questions concerning its complete safety. This is because nanoparticles are generally more soluble and have a better ability to pass through the intestinal wall than larger particles, which in turn may lead to unwanted, harmful effects. It is still not sure if the food-grade TiO_2_ is involved in inducing toxic (e.g., proinflammatory or carcinogenic) processes in humans. Therefore, it is essential to conduct more studies on the toxicity of TiO_2_ NPs, taking into consideration the newest data. It is suggested that the further usage of E171 should be reconsidered, as it offers neither nutritional value nor extended shelf life. If the manufacturers persist in using E171, maximum daily intake levels ought to lead.

Referring to the application of TiO_2_ NPs as a sunscreen component, probably the most significant uncertainty concerns the penetration of these nanoparticles through the outermost layer of the skin, the stratum corneum, and their potential further passage to the bloodstream, which may result in their appearance in biological fluids. A few in vivo studies demonstrate the induction of oxidative stress processes and pathological lesions in different organs, such as the liver. Although the European Commission and FDA provided recommendations for testing and labeling of sunscreens, there is still a lack of official, standardized, binding guidelines for the manufacturers.

We suggest that in the penetration studies of TiO_2_ NPs in UV-filters, scientists should always take into consideration the type of formulation (cream/lotion/oil and type of the emulsion), way of application (cream/spray), the size of NPs and their surface properties. Further studies are necessary to determine whether TiO_2_ NPs passage through the SC and underlying parts of the skin leads to their presence in the bloodstream and the distribution to various organs (Table 3). In recent years, there has been an increasing amount of literature published on the surface modifications of various NPs. It seems that changing surface properties might be the key to obtain TiO_2_ NPs which are biologically inert but effective in terms of UV-blocking. The proper coating might decrease the penetration rate from the skin, as well as the absorption rate from the digestive tract. Noteworthy, developing green synthesis methods may also lead to the improvement of TiO_2_ NPs properties, as well as their stability.

## Figures and Tables

**Table 1:** Examples of in vitro and in vivo studies to assess dermal exposure to TiO_2_ NPs in animals and humans.

References Year	Properties of the Formulation (Type of Emulsion, Size, Structure of TiO_2_ NPs)	Type of Study	Penetration through the SC? Observations
Pelclova et al. [100] 2019	43 nm, oil-free formulation, crystalline structure not specified	in vivo, human participants	Yes Absorption of TiO_2_ NPs through human skin—detectable levels in blood and urine
Zhang et al. [101] 2019	15–40 nm, for in vivo study nano-TiO_2_ solution was dripped on the skin of the mice	in vitro—HUVEC, in vivo—Balb/c mice	not indicated in vitro—increase in ROS and sICAM-1 levels, a decrease in cell viability; in vivo—increase in ROS-dependent markers concentration in mouse serum Protective effects of vitamin E demonstrated
Crosera et al. [102] 2015	38 nm, suspension of commercial TiO_2_ nanopowder dispersed in synthetic sweat	in vitro, human abdominal skin (intact and damaged by needle-abrasion technique)	No No penetration of TiO_2_ NPs in either intact or damaged skin
Xie et al. [103] 2015	20 nm, rod-shaped rutile-type TiO_2_ NPs radiolabeled solution (1 mg/mL)	in vitro, rat skin: intact and slightly damaged with sodium lauryl sulphate (SLS) solution	No No penetration of TiO_2_ NPs in either intact or damaged skin, both in vitro and in vivo
Miquel-Jeanjean et al. [104] 2012	20–30 nm × 50–150 nm, needle-shaped particles, water-in-oil commercial emulsion	in vitro, four specimens of domestic pig ear skin: intact, damaged (stripped), irradiated, damaged and irradiated	No TiO_2_ NPs remained in the uppermost layers of the SC, even if the skin barrier function was impaired
Monteiro-Riviere et al. [105] 2011	10 × 50 nm, mean agglomerates 200 nm; o/w and w/o commercial formulations; rutile	in vitro—skin in flow-through diffusion cells; in vivo—weanling white Yorkshire pig skin	Minimal penetration of TiO_2_ NPs into the upper epidermal layers: in vitro—epidermal penetration, minimal transdermal absorption; in vivo—Ti within the epidermis and superficial dermis, no transdermal absorption detected; UV-B sunburned skin slightly enhanced the SC penetration
Sadrieh et al. [98] 2010	Sunscreen formulation with: uncoated NPs (anatase and rutile): 30–50 nm, coated NPs (rutile): 20–30 nm in diameter and 50–150 in length, submicron particles (rutile): 300–500 nm	in vivo, Yucatan minipig skin	No No structural abnormalities in the skin cells observed
Filipe et al. [97] 2009	Sunscreen (hydrophobic) formulation with: TiO_2_: not indicated TiO_2_ and ZnO: not indicated Coated rutile TiO_2_ material: 20 nm	in vivo, human participants	No Levels of TiO_2_ NPs too low for detection beneath the SC, no toxic effects
Senzui et al. [106] 2009	Rutile TiO_2_ NPs, noncoated and coated; 35, 10 × 100, and 250 nm; 10% cyclopentasiloxane suspension	in vitro, Yucatan micropig skin: intact, stripped and hairless	No No penetration through viable skin, however, TiO_2_ particles penetrated relatively deeply into the skin, possibly via empty hair follicle
Wu et al. [99] 2009	TiO_2_ powders suspensions: anatase: 4 and 10 nm, rutile: 25, 60, 90 nm, anatase/rutile: 21 nm (P25)	in vitro—porcine skin, in vivo—hairless mice	Yes Toxic effects after subchronic exposure
Gontier et al. [107] 2008	Formulations: carbomergel with Degussa P25 (mixture of rutile and anatase, NPs of average size 21 nm, uncoated, approximately spherical platelets), hydrophobic basisgel with Eusolex T-200 (rutile, 20 × 100 nm, coated with Al_2_O_3_ and SiO_2_, lanceolate shape), polyacrylategel with Eusolex T-2000, a commercial sunscreen	Samples of: porcine skin; human skin (dorsal region and buttocks); human skin grafted to SCID-mice	No Porcine skin: TiO_2_ NPs found only on the surface of the outermost SC layer; human skin: penetration of NPs only into 10 μm layer of the SC; human skin grafted to SCID-mice: TiO_2_ NPs attached to the corneocytes
Mavon et al. [108] 2007	Formulation: w/o emulsion containing 3% TiO_2_ NPs with a mean diameter of 20 nm	in vitro—abdominal/face skin from human donors, in vivo—upper arms skin of human donors	No No TiO_2_ NPs detected in the follicle, viable epidermis or dermis. TiO_2_ NPs accumulation in the uppermost layers of the SC (also in opened infundibulum)
Pinheiro et al. [109] 2007	Commercial sunscreen formulation	samples of human skin: healthy and psoriatic, from sacral-lumbal region	No In normal skin, TiO_2_ NPs were retained at the outermost layers of SC, in psoriatic skin, the penetration was slightly facilitated, but in both types of skin, the NPs did not reach living cell layers

Abbreviations: sICAM-1—soluble intercellular adhesion molecule-1; HUVEC—human umbilical vein endothelial cells; ROS—reactive oxygen species; NPs—nanoparticles; SC—stratum corneum.

**Table 2:** Conclusions on the review of the literature regarding the safety of titanium(IV) oxide nanoparticles.

Type of Usage/Application of TiO_2_ NPs	Conclusions	
Food additive E171 ** 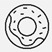 **	The absorption of TiO_2_ from the digestive tract.	Generally considered as extremely low.
	Safety of the long-term dietary exposure to TiO_2_ NPs.	Still uncertain: potential toxic effects may concern the absorption, distribution, and accumulation of TiO_2_ NPs.
	Potential risks caused by TiO_2_ NPs.	Genotoxicity, inflammatory response, carcinogenesis.
	Lack of established, acceptable daily intake limits.	
Sunscreen formulation 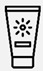	Penetration of TiO_2_ NPs through the SC.	Lack of certainty.
	Generation of ROS on the skin surface and underneath—potential penetration through the skin and harmful effects?	Some evidence on the increase in oxidative stress marker levels has been reported.
	Lack of standardized guidelines for the quality control of sunscreens.	

**Table 3:** Most probable perspectives in future studies regarding the safety of titanium(IV) oxide nanoparticles in everyday products.

Type of Usage/Application of TiO_2_ NPs	Perspectives
Food additive E171 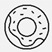	Conducting a thorough safety assessment of E171 (especially its nanofraction).
	Developing surface modification methods (e.g., to decrease the absorption rate) as well as green synthesis technologies.
	Establishing E171 daily intake levels considering different particle sizes, polymorphic structures, surface modifications, etc.
Sunscreen formulation 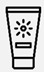	Providing complete assessment of the risk associated with the usage of TiO_2_ NPs-containing sunscreens.
	Improving the action of TiO_2_ NPs by their surface modification and green synthesis.
	Providing official guidelines for sunscreen manufacturers.
